# High-quality DNA isolation protocol for detection of Khapra beetle (Dermestidae: *Trogoderma granarium* Everts, 1898) in standard wheat germ trap

**DOI:** 10.1007/s11033-023-08673-1

**Published:** 2023-08-04

**Authors:** Xiaocheng Zhu, David Gopurenko, Francesca Galea, Ian B. Marsh, Sandra McDougall, Agasthya Thotagamuwa

**Affiliations:** 1 NSW Department of Primary Industries, Wagga Wagga Agricultural Institute, Wagga Wagga, NSW 2650 Australia; 2 NSW Department of Primary Industries, Elizabeth Macarthur Agricultural Institute, Menangle, NSW 2568 Australia; 3 NSW Department of Primary Industries, Yanco Agricultural Institute, Yanco, NSW 2703 Australia; 4 School of Agriculture, Environment and Veterinary Sciences, Charles Sturt University, Wagga Wagga, NSW 2650 Australia

**Keywords:** Quantitative PCR, Grain pest, DNA extraction, Molecular identification, Khapra beetle, *Trogoderma granarium*

## Abstract

**Background:**

Khapra beetle (Dermestidae: *Trogoderma granarium* Everts, 1898) is an internationally significant pest of grain crops and stored grain products. Wheat germ traps, routinely used in surveillance sampling of Khapra beetle provide feed-substrates used by the pest throughout its life cycle. However, Khapra beetle larvae, eggs and other traces of the pest, such as larval frass and exuviae, in wheat germ traps are difficult to sort and taxonomically identify. Additionally, high levels of polysaccharides in wheat germ can inhibit PCR based molecular detection of this pest captured in the traps.

**Methods and results:**

We have developed a sensitive and low-cost protocol for extracting trace levels of Khapra beetle DNA from an entire wheat germ trap. Overnight digestion of entire trap contents in 6 mL of ATL buffer, followed by a 40 min lysis step was optimal for DNA extraction. Paired with reported qPCR assays, this protocol allows the detection of a few hairs of *T. granarium* in a typical 2-gram wheat germ trap.

**Conclusion:**

This DNA extraction protocol makes it possible to perform a more rapid identification of the pest following wheat germ sample collection. The protocol has potential to improve international efforts for Khapra beetle surveillance.

## Introduction

Khapra beetle (Dermestidae: *Trogoderma granarium* Everts, 1898) is native to tropical south Asia and is one of the world’s most significant pests of grain products [1]. In Australia, it is listed as the number one priority pest for grains and the second highest priority plant pest [2]. *Trogoderma granarium* is not present in Australia but interceptions at ports of entry have been reported [3] that pose a significant biosecurity threat to Australian grain industries [4]. Fortunately, incursions have been contained and eradicated to date. *Trogoderma granarium* can infest over 100 commodities and the contaminated products can cause gastrointestinal problems if ingested by humans [4]. An outbreak in Australia could result in $15.5 billion lost over 20 years [5].

*Trogoderma granarium* spends most of its life at the larval stage. Under ideal conditions, the species can complete its lifecycle in 26 days [6]. However, larvae can enter diapause state which can last for six years or longer [1, 3]. Gravid female adults lay up to 100 un-clustered eggs across grain substrates. Eggs are typically less than 1 mm in length and not readily apparent by casual observation [4]. Suspected infestations by the pest are typically first noted when concentrated presence of larval frass and exuviae is observed. Conversely, short lived adults may rarely be seen.

Infield determination of Khapra beetle is extremely complicated, owing to its similarity to other Dermestid beetles. Identification of suspected Khapra beetle specimens and/or their trace tissues requires specialist taxonomic support. Traps designed for capture of *T. granarium* include sex attractant pheromone traps and food-bait wheat germ traps [1]. Pheromone traps attract short lived male adults. In contrast, wheat germ traps allow for complete life-cycle and long-term sampling of the pest by providing a substrate suitable for egg deposition, larval development, and larval diapause [7]. A typical wheat germ trap is a 5 cm (W) × 1.5 cm (D) × 1 cm (H) plastic tray filled up to 1/3 full with wheat germ [1], which normally weigh less than 2 g. Wheat germ traps are inspected for evidence of beetle presence (adults, larvae, eggs, exuviae and frass) and microscopically examined for taxonomic identification, or in the case of eggs, reared out for later stage identifications. The whole process of sorting specimens and trace tissues in wheat germ traps is painstakingly time consuming and labour intensive. Furthermore, taxonomic identification of pre-adult instar specimens and degraded adult samples is challenging. Alternatively, rapid molecular techniques, such as Loop-mediated isothermal amplification (LAMP) [8] and qPCR [9–11], reported for Khapra beetle identification are extremely useful when species identification from trace tissues is uncertain or impossible [11].

LAMP and qPCR are routinely used for detection and or quantification of target insect DNA in environmental samples [11–13], including dust samples and wheat germ. This can potentially alleviate the labour-intensive needs for sorting and rearing of specimens. However, it is difficult to obtain high quality DNA from environmental samples whilst excluding inhibitory substances. Most commercially available DNA extraction kits are designed for low volume sample processing. Extraction of larger sample volumes from a standard wheat germ trap would require significant amounts of reagents costing upwards of $50 per extraction. In addition, commercial kits are normally not suitable for DNA extraction from samples containing high levels of polysaccharides and/or proteins [14], such as is present in wheat germ. Wheat germ polysaccharides interact with DNA to form a highly viscous solution unsuited to filtering through a DNA extraction column. The national diagnostic protocol for Khapra beetle (NDP) includes several DNA extraction protocols, however, all of which were designed for extraction from individual insect specimens [11]. Trujillo-González, et al. [13] developed a DNA extraction protocol for *T. granarium* from dust samples by fractioning the samples followed by extraction using a DNeasy Blood & Tissue Kit (Qiagen). A concern for any extraction technique is that more PCR inhibitors are being extracted when larger quantity of samples are used. In wheat germ, for example, there is large amount of polysaccharides that inhibits the downstream PCR [15]. Several DNA extraction protocols were developed for *Tribolium* beetle species in wheat flour [16, 17] or oat flakes [18]. However, these protocols were tested using either homogenised insect [16, 18] or insect DNA [17] added into flour or oat flakes to simulate contaminated environmental sample. Such an approach might provide a reliable indication of analytical sensitivity however true diagnostic sensitivity can only be determined by including un-homogenised tissue of a target species in the sample background. Here, we have used this approach to simulate uneven distribution of the target.

Given the utility of wheat germ trapping of Khapra beetle over its life cycle, a reliable DNA extraction method is required to obtain assayable levels of trace insect DNA from wheat germ. First, we used homogenised *T. granarium* tissue to contaminate wheat germ to develop an optimal DNA extraction protocol, which was then tested against non-homogenised *T. granarium* tissues. This low-cost protocol will allow for retrospective examination of genetically detected *T. granarium* recovered from traps.

## Materials and methods

### Insect and wheat germ materials

All insects used in this study were ethanol preserved dead specimens from quarantine interceptions. They were taxonomically identified [11] and provided by the Australian Insect Collections unit, Orange Agriculture Institute, NSW Department of Primary Industries. The identity of the specimens was confirmed by COI DNA barcode sequence (GenBank accession number: OP597471-OP597472). Three *T. granarium* larvae were homogenised in liquid nitrogen using mortar and pestle. Then the homogenised larvae were resuspended with 650 µL of ATL buffer (Qiagen, Australia). A total of 600 µL of the homogenised larvae was collected and stored under − 20 °C until extraction.

A standard wheat germ trap used in surveillance for *T. granarium* typically consists less than 2 g of raw wheat germ (Lotus, KADAC PTY LTD, Australia) [6]. In our experimental trials, replicate wheat germ samples weighing two grams (2.05 ± 0.02 g) were each spiked with 5µL of homogenised *T. granarium* larvae (approximately equal to 2% of the body mass of final instar larva). The contaminated wheat germ samples were then subjected to DNA extraction. Wheat germ samples free of larval homogenate were used as negative controls for DNA extraction and downstream qPCR. In addition, to confirm the extraction protocol also performed well with non-homogenised tissues, we mixed 2 g of wheat germ with a trace amount of larval tissues including a small piece of skin (< 1mm^2^) and a small amount (< 5) of hairs taken from larva.

### DNA extraction

DNA extractions through spin columns were optimised for yield and quality of DNA using trial modifications to protocols and quantities of commercially available buffers used in DNeasy Blood & Tissue Kit (Qiagen, Australia). Quantities of proteinase K and ATL buffer, and times for sample digestion in ATL buffer and for lysis in AL buffer, were tested for their impacts on the yield and quality of extracted DNA. The experimental design consisted of 3 proteinase K concentrations × 2 ATL buffer amounts × 2 digestion times × 3 lysis times × 3 replications = 108 replicate digestions prepared for DNA extractions (Table 1). Replicates contained contaminated wheat germ sample loaded into a 50mL conical tube and well mixed with proteinase K (25, 50 or 100µL at 20 mg/mL, Qiagen, Australia) and ATL buffer (6 or 10mL, Qiagen, Australia). The replicates were incubated in an orbital shaking incubator (RATEK, Australia) at 56 °C and 200 rpm for four hours or overnight. After incubation, digestions were centrifuged at 2,250 g for five minutes. A millilitre of supernatant was transferred to a new microtube and centrifuged again at maximum speed (> 10,000 g) for five minutes. After that, 200µL of supernatant was carefully transferred to a new microtube and any precipitate was avoided. Equal amount of AL buffer (Qiagen, Australia) was added to the supernatant, mixed and incubated under 56 °C for 10, 20 or 40 min. After incubation, 200µL of absolute ethanol was added to the mixture and mixed thoroughly by vortexing. Then the entire volume was passed through a spin column (EconoSpin, Epoch lifescience, USA) at 10,000 g for 3 min or until all the liquid had passed through. Finally, DNA was washed with 500µL of AW1 and AW2 buffer (Qiagen, Australia) and eluted with 100µL of AE buffer (Qiagen, Australia).

**Table 1:** Tested extraction conditions in this study, including amount of proteinase K, amount of ATL buffer, time of digestion and time of lysis

Proteinase K (µL)	ATL buffer (mL)	Time of digestion	Time of lysis (min)
25	6	4 h	10
50	10	Overnight	20
100			40

### DNA quantity and quality assessment

The quantity and quality of extracted DNA was checked using optimised Khapra beetle detection II qPCR assay targeted to the mitochondrial NADH dehydrogenase subunit 6 (*ND6* gene) region (Table 2) [9]. Reactions were carried out in a 10µL mixture containing 1 × PerfeCTa qPCR ToughMix (Quantabio, USA), 0.3µM forward primer, 0.9µM reverse primer, 0.15µM probe and 2.5µL DNA template. The qPCR cycle started with an initial denature at 95 °C for 3 min, followed by 40 cycles of denaturation (95 °C for 10 s) and anneal-extension (60 °C for 15 s). A novel 180 bp double stranded gBlock fragment designed here based on Khapra beetle ND6 sequence (GenBank accession NC_053875.1) was used in estimation of a standard curve (Efficiency: 0.968 and R^2^: 0.999) for calculation of qPCR amplified KB DNA concentration. QPCR reactions were conducted on a Magnetic Induction Cycler PCR machine (Bio Molecular Systems, Australia). DNA template concentrations and qPCR efficiency of each reaction were calculated using associated Bio Molecular Systems software.

**Table 2:** qPCR primers, probe and gBlock sequences used in this study. The primers and probe were designed by Furui, et al. [9]; the gBlock was newly designed. All oligos were synthesised by Integrated DNA Technologies

Primers	Sequence (5’-3’)
KBII-F	CAGCCTTATATGACTTCTCATACC
KBII-R	GATTTCATGTTGGGAATGATG
KBII-P	5Cy5-GCAAATGGTGGCGAGTGTTGTC-3IAbRQSp
gBlock	AGTAGCATCCAATGAAAAATTCAAATTCTCAATTAAAATCAGCCTTATATGACTTCTCATACCATTGACAACACTCGCCACCATTTGCTTAAATATCAATTCATCATTCCCAACATGAAATCAAGAATCACTTCCAATAGACTTTATCACCCAAACAAACAAATCAATATCAAAATTCAT

### Sensitivity test with non-homogenised tissue

We tested if our protocol was effective on traps containing non-homogenised tissue, and further, if testing could be conducted within a single day. Wheat germ traps with non-homogenised larval tissues were digested for 4 h at 56 °C used 50µL of proteinase K and 6mL of ATL buffer, followed by incubation at 56 °C with AL buffer for 40 min. All other steps in the digestion, DNA extraction and qPCR and product quantifications (obtained from three replicates measures per digestion) are as reported in our primary protocol.

### Data analysis

All data analyses were performed in R version 4.04 [19]. The impacts of proteinase K and ATL buffer, digestion time and lysis time on the yield of DNA and qPCR efficiency were evaluated using ANOVA with R packages tidyverse [20] and rstatix [21]. Calculated DNA concentrations were log transformed before the analysis to match the homogeneity of variance assumption for ANOVA. Mean values were pairwise compared using Tukey post-hoc tests. Box plots were created using R package ggpubr [22].

## Results and discussion

**Fig. 1:**
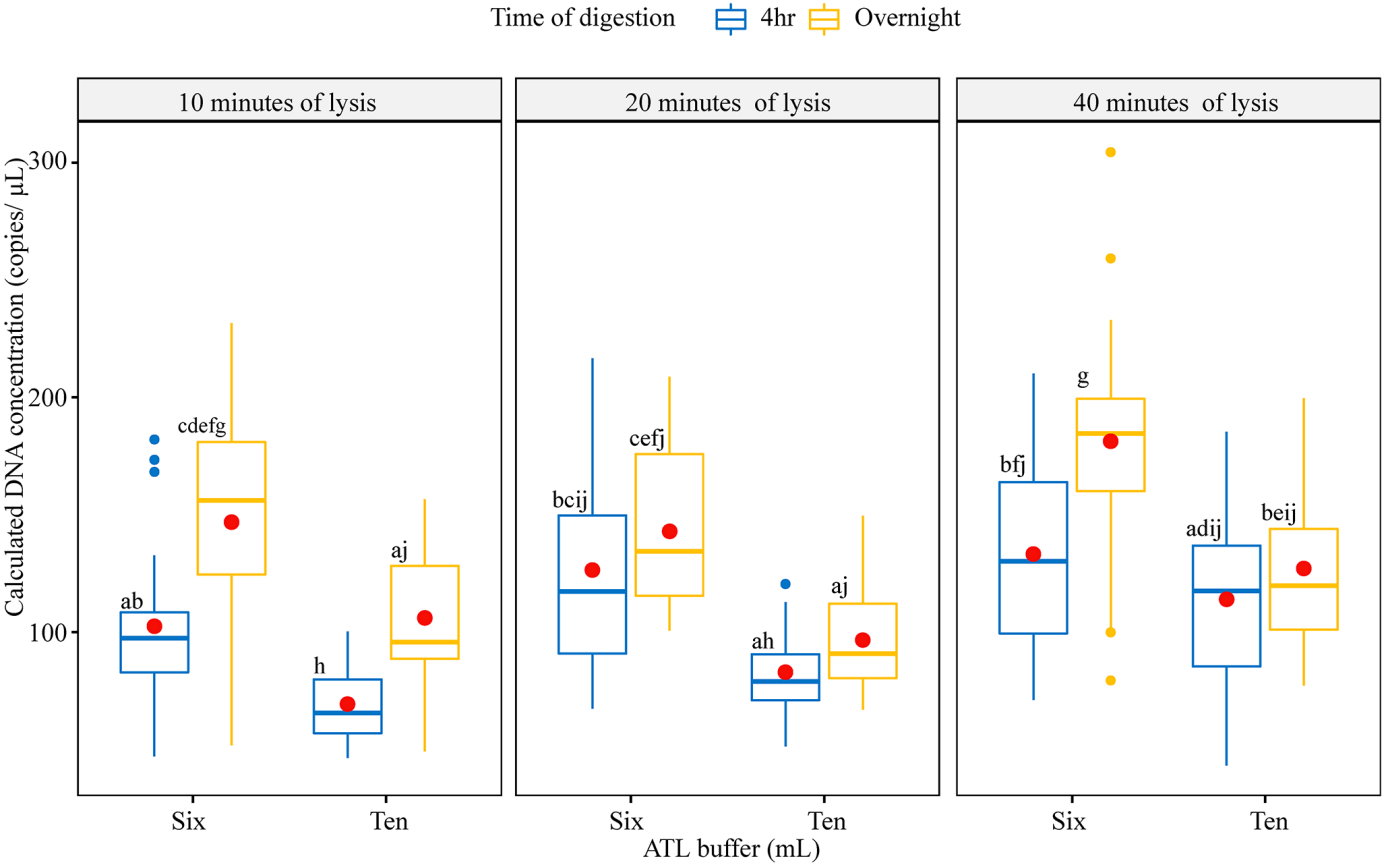
Khapra beetle (*Trogoderma granarium*) DNA extracted from wheat germ trap with various DNA extraction protocols. In the box plots, the boundaries of the box indicate the 25th and 75th percentiles, a thick line within the box marks the median and a red dot within the box marks the mean. Whiskers above and below the box indicate the 10th and 90th percentiles. Points above and below the whiskers indicate outliers outside the 10th and 90th percentiles. There was no significant difference among means sharing the same letter according to Tukey post-hoc tests (*P* < 0.05)

*Trogoderma granarium* DNA was detected in all extraction protocols with Cq (quantification cycle) values ranging from 28.8 to 31.3 cycles despite the small amount of tissue used for DNA extraction. With respect to extraction efficacy, we found that calculated DNA concentration ranged from 43.2 to 304.4 copies/µl and on average, DNA extraction with 6mL of ATL buffer, digested overnight, followed by 40 min lysis yielded highest DNA (181.2 ± 9.26 copies/µL, Fig. 1). DNA extraction with 10mL of ATL buffer followed by 4 h of digestion and 10 min lysis yielded least DNA (69.2 ± 2.89 copies/µL, Fig. 1).

All factors, except the quantity of proteinase K, significantly impacted the yield of *T. granarium* DNA in wheat germ trap (Table 3). We observed a significant two-way interaction between time of digestion and time of lysis (*P* = 0.02). In general, longer digestion time coupled with use of less ATL lysis buffer yielded more DNA (Fig. 1). Longer digestion possibly released more PCR inhibitors present in wheat germ as observed from the reduction of qPCR efficiency with the overnight digestion compared to four hours of digestion (0.975 vs. 0.959, *P* < 0.001). Although statistically significant, such a small difference in efficiency is negligible and unlikely to practically impact the detection of *T. granarium* DNA.

**Table 3:** ANOVA results showing the significance of factors and factorial interactions on DNA extraction concentration. Amount of proteinase K did not significantly impact DNA yield, subsequently its interactions with other factors are not shown here. DFn: the degree of freedom for the numerator of the F ratio, DFd: the degree of freedom for the denominator, ***P < 0.001 and * *P* < 0.5

Factors	DFn	DFd	F	*p*
time of digestion (TOD)	1	288	61.206	9.82E-14***
amount of ATL buffer (AAB)	1	288	105.539	2.68E-21***
time of lysis (TOL)	2	288	28.257	6.23E-12***
amount of proteinase K	2	288	0.363	0.696
TOD: AAB	1	288	0.226	0.635
TOD: TOL	2	288	3.95	0.02*
AAB: TOL	2	288	1.142	0.321
TOD: AAB: TOL	2	288	1.292	0.276

The protocol is easily undertaken and reproducible. If a single day turnaround is required, the preparative DNA digestion time can be reduced to 4 h to extract sufficient levels of DNA (133 ± 7.66 copies/µL) for qPCR detection of the pest. In addition, the sensitivity of the qPCR assay could be further increased by using a digital PCR system [23]. In contrast to the protocols reported in the NDP for DNA extraction directly from recovered specimens [11], our protocol was designed to eliminate the labour intensive needs to recover specimens from traps prior to their extraction. Further, the cost of DNA extraction by our protocol is approximately A$10 using proprietary buffers and reagents, and less than an estimated A$50 required if using commercial kits to process partitioned wheat germ traps.

Unlike mock experimental samples spiked with homogenised insect tissue [16, 17], targeted pest tissues in real-world samples are likely to be unevenly distributed. An earlier DNA method for grain pest detection in oak flake traps employed total sample homogenisation prior to DNA extraction [18]. However, in the case of wheat germ traps, this would release large amounts of non-target DNA and PCR inhibitors such as polysaccharides. Subsequently we avoided wheat germ homogenisation in our DNA extraction protocol to minimise presence of these confounding inhibitors during the critical qPCR pest detection step.

To further remove inhibitors, we applied two centrifuge steps at high speed to precipitate protein and wheat germ debris, and another long centrifuge step to allow all viscous lysate wastes to pass through the silica membrane used in DNA capture. The duration of centrifuge can be increased if any viscous mass remains. Any residues on the silica membrane will impact downstream clean up steps.

In our trials, non-homogenised *T. granarium* skin and hair in wheat germ were all detected in the qPCR test. Skin tissues yield more DNA (101 ± 9.5 copies/µL (cq = 30.9)) compared to trace hairs (measured as 6 ± 1.7 copies/ µL (cq = 35.1)). Non-homogenization of trap material allows an added benefit for retrospective examination of wheat germ trap contents, when there is a need to taxonomically corroborate presence of *T. granarium* following DNA detection of the pest.

Our study measured the DNA quantity by qPCR, which could be impacted by PCR inhibitors and extensive wheat germ DNA. Consequently, the measured DNA quantity might be less than actual extracted *T. granarium* DNA. Importantly, all treatments were conducted with same amount of wheat germ to reduce the error among the treatments. On a practical note, our examination (data not reported here) indicates upper limits to the testable volume of wheat germ examined under buffer quantities reported in our protocol. Volumes of wheat germ greater than 3 g lead to total absorption of available buffer, negating any possibility of viable DNA extraction and subsequent qPCR testing.

Due to the current regulation, we are not able to obtain *T. granarium* eggs. However, the amount of tissues used in the extraction were much smaller compared to a single *T. granarium* egg. Therefore, we believe this DNA extraction protocol will also work well with egg detection, which will significantly reduce the requirement of rearing out of wheat germ trap.

## Conclusion

Our study developed a low-cost and high-quality DNA extraction protocol which allows qPCR detection of *T. granarium* trace tissues in standard 2-gram wheat germ traps. According to our qPCR results, overnight digestion of wheat germ trap contents with 6mL of ATL buffer, followed by 40 min lysis in AL buffer provided optimal release of DNA from trace amounts of *T. granarium* tissue. Our protocol eliminates time expensive handling procedures to sort out and identify Khapra beetle traces from wheatgerm traps. This protocol has great potential for application in future emergency responses.

## Data Availability

Khapra beetle COI sequences are deposited to GenBank under accession number OP597471-OP597472.

## References

[1] Stibick J (2007). New Pest Response Guidelines: Khapra Beetle. (USDA–APHIS–PPQ–Emergency and Domestic Programs.

[2] Department of Agriculture Water and the Environment (2019) National Priority Plant Pests (2019). https://www.awe.gov.au/biosecurity-trade/pests-diseases-weeds/plant/national-priority-plant-pests-2019

[3] Plant Health Committee (2021) National Khapra Beetle Action Plan 2021–2031. Department of Agriculture, Water and the Environment. www.awe.gov.au/biosecurity-trade/pests-diseases-weeds/plant/national-action-plans

[4] Department of Agriculture Water and the Environment (2022) Khapra beetle (*Trogoderma granarium*). https://www.awe.gov.au/sites/default/files/documents/khapra-beetle-pest-bulletin.pdf

[5] Department of Agriculture Water and the Environment (2020) Khapra beetle – the story so far. https://www.awe.gov.au/biosecurity-trade/pests-diseases-weeds/plant/khapra-beetle/khapra-beetle-story

[6] Animal and Plant Health Inspection Service (2022) Khapra beetle program manual. U.S. Department of Agriculture. https://www.aphis.usda.gov/import_export/plants/manuals/domestic/downloads/khapra-beetle.pdf

[7] Kataria R, Kulkarni N (2017). Evaluation of a push-pull approach for *Trogoderma granarium* (Evert) using a novel dispensing system for repellents/attractants under laboratory conditions. J Entomol Zool Stud.

[8] Rako L (2021). A LAMP (loop-mediated isothermal amplification) test for rapid identification of Khapra beetle (*Trogoderma granarium*). Pest Manage Sci.

[9] Furui S, Miyanoshita A, Imamura T, Minegishi Y, Kokutani R (2018). Qualitative real-time PCR identification of the khapra beetle, *Trogoderma granarium* (Coleoptera: Dermestidae). Appl Entomol Zool.

[10] Olson R, Farris R, Barr N, Cognato A (2014) Molecular identification of *Trogoderma granarium* (Coleoptera: Dermestidae) using the 16s gene. J Pest Sci 87. 10.1007/s10340-014-0621-3

[11] Byrne O et al (2022) National Diagnostic Protocol for Khapra beetle – *Trogoderma granarium* Everts. Department of Agriculture, Fisheries and Forestry, Canberra City ACT, Australia, p 99

[12] Schneider J (2016). Detection of invasive mosquito vectors using environmental DNA (eDNA) from water samples. PLoS ONE.

[13] Trujillo-González A et al (2022) Detection of Khapra beetle environmental DNA using portable technologies in Australian biosecurity. Front Insect Sci 2. 10.3389/finsc.2022.795379 10.3389/finsc.2022.795379 PMC10926498 38468794

[14] Marín DV, Castillo DK, López-Lavalle LAB, Chalarca JR, Pérez CR (2021). An optimized high-quality DNA isolation protocol for *Spodoptera frugiperda* J. E. smith (Lepidoptera: Noctuidae). MethodsX.

[15] Furukawa K, Bhavanandan VP (1983). Influences of anionic polysaccharides on DNA synthesis in isolated nuclei and by DNA polymerase alpha: correlation of observed effects with properties of the polysaccharides. Biochim Biophys Acta.

[16] Balasubramanian A, Jayas DS, Fernando D, Li G, White NDG (2007) Sensitivity analysis of DNA fingerprinting technique for detecting insect fragments in wheat flour. Can Biosystems Eng / Le Genie des biosystems au Can 49: 4.1–4.5.

[17] Kamel A, Aziz M, Ramadan R (2016) Revealing of wheat products contamination with flour beetles *Tribolium* spp. by molecular technique. IOSR J Biotechnol Biochem 2:81–87

[18] Nowaczyk K (2009). Molecular techniques for detection of *Tribolium confusum* infestations in stored products. J Econ Entomol.

[19] Team RC (2021) R: a language and environment for statistical computing. R Foundation for Statistical Computing. http://www.R-project.org/

[20] Wickham H (2019). Welcome to the tidyverse. J Open Source Softw.

[21] Kassambara A (2021) Rstatix: Pipe-Friendly Framework for Basic Statistical Tests. R package version 0.7.0 https://CRAN.R-project.org/package=rstatix

[22] Kassambara A (2020) ggpubr: ‘ggplot2’ Based Publication Ready Plots. R package version 0.4.0. https://CRAN.R-project.org/package=ggpubr

[23] Taylor SC, Laperriere G, Germain H (2017). Droplet Digital PCR versus qPCR for gene expression analysis with low abundant targets: from variable nonsense to publication quality data. Sci Rep.

